# The summer school students’ viewpoints about important factors in learning, Shiraz University of Medical Sciences

**Published:** 2014-04

**Authors:** LEILA BAZRAFCAN, FARIBA HAGHANI, NASRIN SHOKRPOUR

**Affiliations:** 1 Quality Improvement in Clinical Education Research Center, Shiraz University of Medical Sciences, Shiraz, Iran;; 2 Medical Education Research Center, Isfahan University of Medical Sciences, Isfahan, Iran;; 3 English Department, Paramedical School, Shiraz University of Medical Sciences, Shiraz, Iran

**Keywords:** Learning, Students, Viewpoints

## Abstract

**Introduction**: The main goal of education is learning and change in behavior which has been revolutionized in the 21st century due to the rapid and widespread changes in science. The traditional approach to education does no longer meet the learners' needs, necessitating new changes in educational curricula. This study was designed to determine the factors influencing learning in the 21st century and find out the students' viewpoints on this issue.

**Methods**: This is a descriptive study aiming at determining the students' views on new approaches to learning in the 21st century. To do so, a researcher-made questionnaire was designed. It contained 30 questions in 3 sections including demographic data, background questions and two open questions about their suggestions and criticisms. The reliability and validity of the questionnaire was pilot-tested and measured, which proved to be describable. 150 students participating in university summer schools in Shiraz University of Medical Sciences were enrolled. The questionnaires were sent to the students in person and through electronic mails. The students were asked to return the completed questionnaires to the given email address. The data were analyzed in SPSS, version 14, using descriptive statistics of frequency, mean, percentage and standard deviation and t-test. P<0.05 was considered as statistically significant.

**Results**: 150 questionnaires were appropriately filled out and given to the researchers. The results indicated that, according to the students, 6 factors including the use of computer in teaching, enhancement of virtual learning, the use of mobile in relations, enjoyment of electronic learning contexts, the learning focus on attitudes and the facilitating role of the lectures were the most influential factors in learning. On the other hand, the government's responsibility and responsiveness, creativity and risk taking, increase in the social relationship among the learners, focus on practical skills, and management were considered as the least influential factors in learning in the 21^st^ century.

**Conclusion**: It seems that the students philosophically tend to approve constructivism and cooperative learning which is learner-centered as compared to conventional education which is teacher-contended. According to experts, this type of viewpoint is in the same line with new approaches to teaching and education in the present era. Moreover, it impacts the reforms, complementation and expansion of methodology greatly.

## Introduction

Rapid worldwide changes at the present century have brought about new challenges in education (1). There is no more appropriate, acceptable and efficient option than education for the modern society for confrontation with these challenges. Education is considered as an essential element for multidimensional and stable development of each society. All those engaged in education expect this process to lead to learning (2).

Learning is considered as the outcome of managed or experimental education which is established in the learner through constant practice, leading to change in behavior. Based on different learning theories, learning at each time, location and in each subject requires specific conditions and skills (3, 4). Consequently, experts  in health care consider learning skills in the 21st century as a collection of skills such as critical thinking, information technology; effective interactive communication; creativity and risk taking; interpersonal skills; personal, social and civil responsibilities; high efficacy; prioritizing; planning and managing; flexibility; compatibility; and self-confidence (5). Some researchers consider main skills such as learning and innovation, cooperation and communication, digital literacy, and life, social and intercultural skills (6). A report on education in the 21st century categorizes these into 3 groups of information and relations, thinking and problem solving, and interpersonal and self direction skills (7). Moreover, internationalization of education is on increase, necessitating learning specific skills for living which seem to be beyond conventional methods (8).

A review of literature shows that researchers and educators have applied different methods of devising 21st century skills. Therefore, some experts do not approve of traditional teaching and learning methods which focus on the passive role of learners and approve of the mere increase in information; they believe that such approaches do not meet the future and present educational need of the learners, and recommend free, creative and critical thinking (9-12). Others emphasize the role of self-direction, self-efficacy, self-regulation and self-assessment on the part of the learners and invite the teachers to apply these principles (13-15). Still some others extol the effective use of information technology and communication, intercultural skills, multicultural literacy, extensive awareness and life technology (16-18). In fact, it can be claimed that education and learning should be based on global peace and coexistence (12, 19, 20).

Some researchers have paid attention to one of the 21st century challenges, i.e. training people who can disseminate peace in the world. They believe that education in the 21st century should be directed towards the culture of peace since it plays a crucial role in this regard. It is necessary to shift the educational goals toward pacific awareness based on love (21).

Another study aimed at a survey of the students and teachers' attitudes toward electronic learning in Maleke Ashtar Technical University using a questionnaire. The results revealed that the teachers have a positive view about electronic education. Their sense of efficacy and self satisfaction was the main reason for their tendency. Independence, teachers’ guidance and multi-media education were the most important factors affecting the students’ attitude toward the impact of electronic learning (22).

Several studies have been conducted in other countries as well as ours on learning, factors effecting it, new approaches to learning, and learners’ attitudes on the influential factors on learning. Considering the fact that learners are at the heart of learning and also taking their active role in acquisition of learning experiences into account, which is the feature of new approaches to learning especially in constructivism, we need to know their attitudes about elements of learning in the 21st century. Therefore, this study aimed at determining the learners’ ideas and attitudes about learning in the 21st century as a factor influencing education in summer schools in Shiraz. 

Education Development Center of Shiraz University of Medical Sciences arranged and held an interdisciplinary educational program containing four subjects of medical education, creativity and genius, medical ethics and management, and communicative skills as the summer school. In this summer course, the main objective was to expand the participants’ insight into general skills such as learning since in modern approaches there is a focus on the creative and active role of the learners. This also helped us recognize which aspects are essential as viewed by the participants (23, 24). Moreover, we selected active subjects from different parts of the country as those who are more concerned with learning. There was also an attempt to compare the participants’ attitudes as to learning.

## Methods

This study was performed on 150 students participating in summer schools in Shiraz University of Medical Sciences (males and females). A questionnaire consisting of 30 questions in 3 sections including demographic data, background questions and two open questions about their suggestions and criticisms was designed. It contained Likert type questions and background questions as to learning context and learners’ features including information and communication skills, thinking and problem solving skills, and life and inter-personal skills. The participants were supposed to respond to each question based on Likert style (complete agree=5, agree=4, relatively agree=3, relatively disagree=2 disagree=1).

The face and content validity of the questionnaire was approved by 15 experts in the field and its reliability was measured through pilot-testing on 30 participants using Cronbach’s alpha, which proved to be 82.8%. The reliability of each part of the questionnaire was as follows: a) learners=88%, b) teachers=79%, c) educational context=93%, and d) total=82%, revealing a high level of reliability as a whole.

The questionnaires were distributed both in person and through electronic mails. The students were asked to fill out the forms carefully and return them.

The data were analyzed in SPSS 14 (SPSS Inc, Chicago, IL, USA), using descriptive statistics of frequency, mean, percentage and standard deviation, one sample t-test, p<0.05 was considered as statistically significant. 

## Results

150 questionnaires were returned and used for analysis of data. Based on the results, there were 91 males (61%) and 59 females (39%). As to their faculty, 41 students (27%) were students of medicine, 18(12%) dentistry, 8(5%) pharmacy , 40(27%) nursing and midwifery, 6(4%) rehabilitation, 22(15%) paramedical sciences, and 2(1%) health and management students. Their mean age was 21±5.9 with 20 years as the youngest and 27 as the oldest students. As shown in Table 1, the factors influencing learning are categorized as students and teachers. The students’ domain showed the highest mean (3.97±0.42). The highest percentage and frequency were related to the use of mobile phone in communications, learners’ motivation, enjoying social learning context, enjoying electronic learning context, enhancing communication among learners, focusing on the role of educators, and the students’ creativity and risk tasking.

The teachers’ domain was the second factor affecting learning (3.97±0.42). Factors as focus on cognitive knowledge focus on practical skills, role of learning in creating attitude, facilitating role of teachers in learning, planning and management showed the highest frequency and percentage.

According to the total percentage and frequency of strongly agree and agree (Table 1), out of 21 factor surveyed, the use of computer in education, development of virtual learning, use of mobile phone in communications, enjoying the learning context, enjoying the electronic learning contexts, focusing on learning to create attitudes, facilitating role of teachers were the most influential factors. On the other hand, governments’ responsibility and responsiveness, creativity and risk taking, development of learners’ social interactions, focus on learning practical skills, management and planning were the least influential factors on learning in the 21st century.

**Table 1:** Comparison of the student's responses as to learning context as a factor impacting learning

**No.**	**Item**	**Mean±SD**	**Mean score of Likert scale**	**p**
**1**	Learning context rich with learning	4.30±0.86	3	<0.001
**2**	Multimedia learning context	4.22±0.81	3	<0.001
**3**	Use of computer in learning	4.45±0.68	3	<0.001
**4**	Development of virtual learning	4.42±0.75	3	<0.001
**5**	Increased educational facilities	3.50±1.26	3	<0.001
**6**	Confidence in electronic learning contexts	4.13±1.06	3	<0.001
**7**	Teaching search strategy	4.31±0.83	3	<0.001
**8**	Government’s responsibility and responsiveness	3.02±1.15	3	>0.05
**Leaning context**		3.70±0.35	3	<0.001

Significance level=0.05

The results of the Table reveals that 4 out of 5 related to the teacher are significantly higher than the average (p<0.01). This shows that the students consider these 4 variables influential in learning. Moreover, they only consider planning and management as moderately influential (p>0.05).

**Table 2:** Comparison of the student's responses to learner domain as a factor impacting learning

** No.**	**Item**	**Mean**±**SD**	**Mean score of Likert scale**	**p **
**1**	Use of mobile phone in communication	4.22±0.85	3	<0.001
**2**	Motivated learners	4.13±1.03	3	<0.001
**3**	Enjoyment in social leaving contexts	4.16±0.97	3	<0.001
**4**	Enjoyment in electronic learning context	4.36±0.77	3	<0.001
**5**	Expansion of social relations among learners	3.40±1.14	3	<0.001
**6**	Creativity and risk larking	3.19±1.43	3	>0.05
**7**	Flexibility	4.49±0.76	3	<0.001
**8**	Teaching life skills	3.78±1.15	3	<0.001
**Learners **		4.02±0.33	3	<0.001

Significance level=0.05

**Table 3:** Comparison of the student's responses to teacher domain as a factor impacting learning

**No.**	**Item**	**Mean**±**SD**	**Mean score of Likert scale**	**p**
1	Focus of learning circumstance	3.81±1.12	3	<0.001
2	Focus on learning practical skills	3.50±1.24	3	<0.001
3	Focus of learning on cheating attitude	4.21±0.92	3	<0.001
4	Facilitating role of teaches	4.53±0.61	3	<0.001
5	Planning and management	3.18±1.24	3	>0.05
**Teaches**	Planning and management	3.85±0.50	3	<0.001

Significance level=0.05

Based on the information in Table 3, the mean score of the students who use chatting is significantly higher than those who do not use it only in “learning environment” (p<0.05).

For the final judgment in the qualitative part of the study, two open questions were used and then the experts analyzed the content of the students' responses.  Then they categorized the students' suggestions on the best facilitators of learning in the 21st century. Based on the content analysis to find the best facilitators by experts, the reasons were categorized into four groups of technology, self efficacy, learner interest, and good communication. According to the results, 45% of the best facilitators were related to technology, 25% to good communication, 16% to self efficacy, and16% to students’ interest. 

## Discussion

The importance of teaching and learning and its changes in the present era, on one hand, and focus on student-centered strategy and participation of students in learning process, on the other hand, are the significant goals of educational centers and institutes (25).

As to the main research question of this study, the results indicated that students are familiar with elements of learning in the 21st century and know its principles. The mean scores showed that among the 3 factors under the study, the “learners” scores exceeded those of “teachers and educational context”. This shows that the students agree with self efficiency, self direction and self assessment as the 3 important factors of learning in the present century.

Gagatsis et al conducted a study on the students of mathematics to find the effect of self regulation. The first group was taught and supported through self regulation while the second group did not receive any support. Comparison of the results of the pre-test with those of post-test revealed that the students supported for self regulation significantly outperformed the group without such support (26).

Te second priority was given to the role of teacher. To increase self awareness and self efficacy which are necessary for learning in the 21st century, there is a need for teachers to apply new methods of teaching and develop self efficacy and self esteem in them as teachers. According to psychological views, self efficacy means that the individual is confident about his/her capabilities in different teaching circumstances (27, 28).

Moreover, research has shown that the teachers with high level of self efficacy are more likely to apply student-centered methods while incompetent teachers tend to apply teacher-centered approaches.

The results of this study indicated that students approve of the facilitating role of teachers. But what could be the method of guiding students in 21st century? The fact is that the teachers and lecturers are not that trained on the principles and different methods of learning such as interactive methods in small groups and designing and better management of groups, so they cannot apply them in their teaching.

Al-Alawi, in a research on the role of social structure in incapacitating lecturers, found out that characteristics as self respect, distribution of powers, sharing information, knowledge, leadership and organizational culture were influential on their capabilities (29).

The next factors were related to the learning context and facilities. The results indicated that among the 21 factors under the study, the use of computers in teaching, developing virtual learning, using mobile phone in communication, and enjoying the learning contexts were the most effective factors in learning in the 21st century.

These opportunities will surely be provided through modern teaching technologies and electronic learning. Probably, the students are interested in electronic learning since this method can be applied in different ways and provides opportunities for learning based on specific time, location and content or else, the students emphasize the role of context and facilities. However, this cannot alone lead to effective and deep learning (8, 12, 14).

Azevodo and Cromley approved the advantages of protective self-regulation in electronic learning context, which is a necessity in learning in the present century (16). They found out that the teacher, as the individual who is involved in the student’s comprehension constantly, plays a significant role. Furthermore, researchers analyzed verbal learning activities and found out that there is a significant relationship between planned learning contexts, advancement control, and the use of learning strategies in a great number of participants. There are some limitations in this study, including lack of availability of the participants, lack of interest, incomplete responses, and the problems in the files sent by the participants.

## Conclusion

Experts believe that today’s capability requires other efficiencies than traditional and previous capabilities. Now, we do not expect individuals to be just familiar with known problems, and their own workplace and life; rather, they are expected to recognize their own position in future society considering the probable complications and challenges. In this way, they will be able to cope with upcoming issues.

In general, it seems that student's attitude is now based on constructivism and collaboration. Such a viewpoint supports new approaches to teaching and education in the present era and plays a main role in development and reform of teaching methods.

“Interaction” has a vital role in learning and teaching process, creating an opportunity to access knowledge and make worldwide communication possible by exploitation of modern technologies. Therefore, it seems necessary to pay attention to educational programs, a part of which is creative and competent lecturers.

The educational context should be equipped with modern educational technology since it is believed that creative and active adjustments affect the quality of education greatly. Creative adjustments can make a connection between educational curricula and objective and subjective concepts.

Some course contents in an appropriate environment enriched with standards impact the learning efficiently. Therefore, to face the immense and complex changes in the 21st century, we need to take the role of self efficient and self efficacy learners, competent and motivated teachers, and advanced educational context into account. 
